# A Cross-linking Mass Spectrometry Approach Defines Protein Interactions in Yeast Mitochondria

**DOI:** 10.1074/mcp.RA120.002028

**Published:** 2020-04-24

**Authors:** Andreas Linden, Markus Deckers, Iwan Parfentev, Ralf Pflanz, Bettina Homberg, Piotr Neumann, Ralf Ficner, Peter Rehling, Henning Urlaub

**Affiliations:** 1 Bioanalytical Mass Spectrometry Group, Max Planck Institute for Biophysical Chemistry, Göttingen, Germany; 2 Institute of Clinical Chemistry, University Medical Center Göttingen, Göttingen, Germany; 3 Department of Cellular Biochemistry, University Medical Center Göttingen, Göttingen, Germany; 4 Cluster of Excellence “Multiscale Bioimaging: from Molecular Machines to Networks of Excitable Cells” (MBExC), University of Göttingen, Göttingen, Germany; 5 Department of Molecular Structural Biology, Institute for Microbiology and Genetics, Göttingen Center for Molecular Biosciences, Georg-August-University Göttingen, Göttingen, Germany; 6 Max Planck Institute for Biophysical Chemistry, Göttingen, Germany

**Keywords:** Protein cross-linking, mitochondria function or biology, quantification, modeling, yeast

## Abstract

Protein cross-linking and the analysis of cross-linked peptides by mass spectrometry is currently receiving much attention. Not only is this approach applied to isolated complexes to provide information about spatial arrangements of proteins, but it is also increasingly applied to entire cells and their organelles. As in quantitative proteomics, the application of isotopic labeling further makes it possible to monitor quantitative changes in the protein-protein interactions between different states of a system. Here, we cross-linked mitochondria from *Saccharomyces cerevisiae* grown on either glycerol- or glucose-containing medium to monitor protein-protein interactions under non-fermentative and fermentative conditions. We investigated qualitatively the protein-protein interactions of the 400 most abundant proteins applying stringent data-filtering criteria, *i.e.* a minimum of two cross-linked peptide spectrum matches and a cut-off in the spectrum scoring of the used search engine. The cross-linker BS3 proved to be equally suited for connecting proteins in all compartments of mitochondria when compared with its water-insoluble but membrane-permeable derivative DSS. We also applied quantitative cross-linking to mitochondria of both the growth conditions using stable-isotope labeled BS3. Significant differences of cross-linked proteins under glycerol and glucose conditions were detected, however, mainly because of the different copy numbers of these proteins in mitochondria under both the conditions. Results obtained from the glycerol condition indicate that the internal NADH:ubiquinone oxidoreductase Ndi1 is part of an electron transport chain supercomplex. We have also detected several hitherto uncharacterized proteins and identified their interaction partners. Among those, Min8 was found to be associated with cytochrome *c* oxidase. BN-PAGE analyses of *min8*Δ mitochondria suggest that Min8 promotes the incorporation of Cox12 into cytochrome *c* oxidase.

## Abstract (precis)

Protein interactions in mitochondria isolated from *S. cerevisiae* grown on glycerol or glucose medium were analyzed by XL-MS. The non-cleavable cross-linker BS3 proved suitable for elucidating differences in protein-protein interactions under both conditions. XL-MS analysis of interprotein interactions using stable-isotope-labeled BS3 revealed certain limitations in the quantitative application of cross-linking to quite different cellular states. The results from glycerol-grown mitochondria show that Ndi1 interacts directly with CIII in an ETC supercomplex and that Min8 promotes Cox12 assembly into CIV.

## Graphical Abstract

**Figure:**
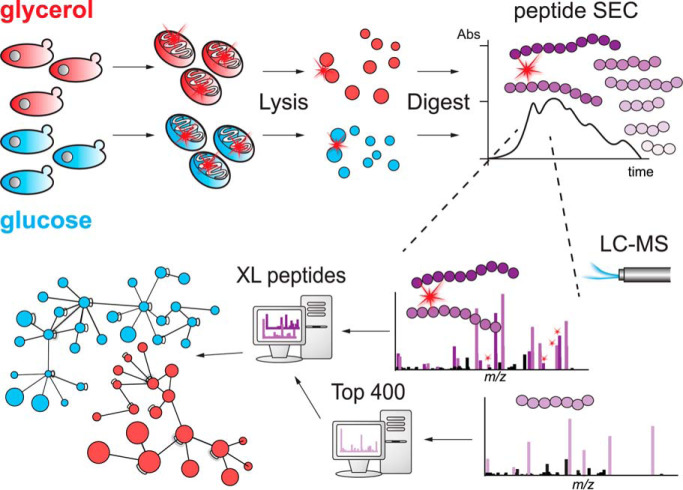

**Highlights**

- XL-MS reveals new PPIs in yeast mitochondria under glycerol and glucose condition.
- Significant but limited results from quantitative XL-MS experiments.
- Ndi1 participates in a CIII_2_CIV_2_ respiratory supercomplex.
- Min8 promotes assembly of Cox12 into an intermediate complex IV.

Mitochondria play key roles in energy production and metabolism of eukaryotic cells. Accordingly, mitochondrial dysfunction has been linked to a variety of human disorders and mitochondria fulfill central tasks in cell fate decisions such as the initiation of apoptosis (1–6). Hence, mitochondrial morphology, biogenesis, metabolism, and protein content have been studied extensively. Mass spectrometry (MS)-based proteomics have allowed the identification, localization, and quantitation of mitochondrial proteins in various species (7–13) and revealed their post-translational modifications (14, 15). Based on these studies, mitochondria contain ∼1000 proteins in yeast (16) and up to 1500 in mammals (17). Most mitochondrial proteins are organized into functional and metabolic networks, *e.g.* protein complexes. Prominent examples for this are the electron transport chain (ETC), the mitochondrial contact-site and cristae-organizing system (MICOS), the translocases of outer and inner membranes (TOM and TIM), the pyruvate dehydrogenase complex (PDH), or multienzyme complexes involved in the tricarboxylic acid cycle (TCA). The underlying protein-protein interactions have been investigated extensively by co-affinity purifications, blue native-polyacrylamide gel electrophoresis (BN-PAGE), or sucrose density gradient centrifugation, revealing large interconnected protein complexes (18–21). These approaches have been complemented with more MS-focused approaches such as APEX (22), BioID (23), or complexome profiling (24) that expanded the number of potential protein-protein interactions, *inter alia* in mitochondria (25–27).

As an alternative approach, chemical cross-linking in combination with MS (XL-MS) has emerged as a powerful tool for the identification of protein-protein interactions (28–31). Here, water-insoluble disuccinimidyl suberate (DSS) and its water-soluble derivative bis(sulfosuccinimidyl)suberate (BS3) are commonly used as protein-protein cross-linkers. Both contain *N*-hydroxysuccinimide (NHS) esters as bifunctional groups that react primarily with the ε-amino group of lysine residues in proteins. XL-MS allows not only the identification of cross-linked proteins that interact with each other but in addition provides the cross-linked peptides and thus topological information. Protein-protein cross-linking has mainly been applied to isolated protein complexes (32–40), where the cross-linking results also allowed to map proteins into the respective 3D structures and to interrogate protein networks. In recent years *in vivo* cross-linking of bacteria, organelles or even entire eukaryotic cells has paved the way for global protein network analyses, by the application of MS-cleavable and enrichable cross-linkers (41–46). In previous studies mitochondria have been a target for XL-MS analyses (47, 48). These studies provided evidence for respirasome formation in the physiological context in mammalian mitochondria revealing supercomplexes of complex I (CI), complex III (CIII) and complex IV (CIV) of the ETC, which had been defined upon solubilization in BN-PAGE experiments (18). Ryl *et al.* (49) emphasized the presence of a multitude of intra-cross-linked proteins in human mitochondria. In a very recent study of yeast mitochondria an enrichable, MS-cleavable and stable-isotope labeled cross-linker was used to investigate protein-protein interactions (50).

Yeast is an established eukaryotic model organism for studying mitochondrial functions (51). Its facultative anaerobic nature allows yeast cells to generate ATP through oxidative phosphorylation in the presence of oxygen, whereas in the presence of glucose ATP can be produced by fermentation (Crabtree effect (52)). This change of metabolism affects the morphology of mitochondria in yeast (53, 54) and also the copy numbers of proteins (55) whose genes are repressed by glucose (56, 57). Applying XL-MS to isolated yeast mitochondria makes it possible (1) to provide a protein interaction network of mitochondrial proteins including those of the outer (OM) and inner membrane (IM), intermembrane space (IMS) and matrix (M), (2) to establish a protein-protein interaction network for yeast growing on different carbon sources and (3) to identify hitherto unknown protein interactions.

In this work, we cross-linked mitochondria derived from yeast grown on either a non-fermentable (glycerol) or a fermentable (glucose) carbon source with BS3. We identified 2100 unique residue-to-residue cross-links in the glycerol and 1787 cross-links in the glucose data set. Hence, we could demonstrate that a water-soluble but non-cleavable cross-linker such as BS3 is suitable for elucidating protein-protein interactions in complex samples. By applying a stable-isotope labeled cross-linking approach, we were able to quantify differences in protein-protein cross-links in mitochondria between the two growth conditions. However, the significance of this quantitative approach was limited because of high numbers of intraprotein links and different protein copy numbers induced by glucose-repression. The analysis of mitochondria derived from yeast grown on glycerol medium shows that Ndi1, the internal NADH:ubiquinone oxidoreductase, is directly associated to the CIII-CIV supercomplex of the yeast's ETC. Furthermore, we identified the so far uncharacterized protein Min8 as a new constituent of CIV.

## EXPERIMENTAL PROCEDURES

### 

#### 

##### Yeast Strains and Growth Conditions

Yeast strains used in this study are derivatives of *S. cerevisiae* strain YPH499. Deletion of *MIN8* (*YPR010C-A*) was achieved by homologous recombination of a *HIS3MX6* cassette into the corresponding locus. Generation of HA-tagged strains was performed by chromosomal integration. Yeast cells were grown on rich medium (1% yeast extract, 2% peptone) supplemented with 2% glucose, 3% glycerol or 3% lactate. For growth tests, yeast cells from liquid cultures were adjusted to an OD_600_ of 1 and serial dilutions of the culture were spotted onto agar plates containing either glucose, glycerol or lactate as a carbon source and incubated at indicated temperatures. Mitochondria were isolated as previously described from yeast cells grown at 30 °C (58). Mitochondria used for mass spectrometric analyses of BS3 cross-linked proteins were further purified by tandem sucrose gradient centrifugation.

##### Mitochondrial Oxygen Consumption

Oxygen consumption was assessed using high-resolution respirometry (Oxygraph-2k, Oroboros Instruments, Innsbruck, Austria) in 2 ml of respiration buffer (225 mM Sucrose, 75 mm Mannitol, 10 mm Tris, 10 mm KH_2_PO_4_, 5 mm MgCl_2_, 10 mm KCl, pH 7.4) at 30 °C. Non-phosphorylating respiration (LEAK) was addressed using pyruvate (10 mm) and malate (2 mm). Adding ADP in a saturating concentration (1 mm, State3) followed by succinate (10 mm) determines the maximal capacity for oxidative phosphorylation (OXPHOS). Respiration was killed by addition of Antimycin A (5 μM) to prevent electron transfer from CIII to CIV and ascorbate (2 mM) followed by TMPD (N,N,N′,N′-Tetramethyl-p-phenylenediamine; 500 μM) were added to address OXPHOS capacity via CIV. To distinguish between respiration and auto-oxidation of TMPD/ascorbate, NaN_3_ (100 mM) was added to block the O_2_ binding site of CIV and the values were subtracted from the values after TMPD/ascorbate addition.

##### In Vitro Import into Mitochondria

Cox13 and Cox12 were amplified using appropriate primers including a 3′ overhang for SP6 recognition. PCR products were used for *in vitro* transcription. Respective proteins were synthesized in rabbit reticulocyte lysate (Promega, Madison, Wisconsin) in the presence of [^35^S] methionine. For assembly analyses, isolated mitochondria were incubated with radiolabeled proteins in import buffer (250 mm sucrose, 10 mm MOPS/KOH pH 7.2, 80 mm KCl, 2 mm KH_2_PO_4_, 5 mm MgCl_2_, 5 mm methionine, and 3% fatty acid-free BSA; import buffer for Cox12 without BSA), supplemented with 5 mm creatine phosphate and 0.1 mg/ml creatine kinase, in the presence of 2 mm ATP and 2 mm NADH for indicated times. Cox13 reactions were stopped on ice by dissipation of the membrane potential with 8 μm Antimycin A, 1 μm Valinomycin and 20 μm Oligomycin. For Cox12 assembly experiments the reactions were stopped by adding 50 mm iodoacetamide (IAA). All Samples were lysed in 0.6% n-Dodecyl β-d-maltoside (DDM) buffer for BN-PAGE.

##### Immunoprecipitation

Isolation of cross-linked adducts were performed in WT and Min8^HA^ expressing strains. For co-immunoprecipitation, Cox12-specific antisera as well as non-binding Pam18-antisera were bound to protein A-Sepharose (GE Healthcare, Chicago, Illinois). BS3 cross-linked and non-treated mitochondria were lysed in 20 mm Tris (pH 7.4), 80 mm NaCl, 0.5 mm EDTA, 0.5% Triton X-100, 0.1% SDS, 10% glycerol and 1 mm PMSF for 1 h at 4 °C. Lysates were cleared at 20,000 × *g* for 15 min at 4 °C and total samples were taken. Solubilized proteins were incubated for binding with Cox12, Pam18 and HA antibody-columns for 1 h at 4 °C. After intensive washing, the bound proteins were eluted by the application of 0.1 m glycine pH 2.8 and further analyzed by SDS-PAGE and Western blotting.

##### Cross-linking of Purified Mitochondria

Purified mitochondria of each condition were aliquoted in 1 mg portions and stored at −80 °C before use. Aliquots were thawed gently and spun down with 10,000 × *g* for 5 min at 4 °C. Bis(sulfosuccinimidyl)suberate (BS3, non-weight format, Thermo Fisher Scientific, Waltham, Massachusetts) was used as chemical cross-linker and resuspended in cross-linking buffer (10 mm HEPES, pH 7.5, 100 mm NaCl) to a concentration of 100 mm. After diluting the BS3 stock solution to 5 mm, pelleted mitochondria were resuspended in 200 μl cross-linking buffer including BS3 and incubated for 1 h at room temperature (RT) with gentle rotation. The reaction was quenched, and mitochondria were lysed by adding Tris, pH 8, to a final concentration of 50 mm and SDS to 2%. Proteins were boiled at 70 °C for 10 min and after chilling to room temperature precipitated by adding ice-cold acetone four times the sample volume, incubated at −80 °C for 2 h. This was repeated once with different isolations of mitochondria, resulting in two biological replicates per condition.

For the comparison of BS3 with disuccinimidyl suberate (DSS), freshly prepared crude mitochondrial extract derived from yeast grown on glycerol medium was treated as described above with the following changes: isotopically labeled BS3-d4 (non-weight format, Thermo Fisher Scientific) and DSS (non-weight format, Thermo Fisher Scientific, resuspended in DMSO) were mixed in an equimolar ratio and used for the cross-linking reaction. The final concentration of each cross-linker was 1 mm.

For the quantitation, purified mitochondria from yeast grown on glycerol and glucose, respectively, were cross-linked with BS3 and isotopically labeled BS3-d4 (non-weight format, Thermo Fisher Scientific) in a label-swap experiment. Therefore, 0.5 mg of each condition were cross-linked with either 5 mm BS3 or BS3-d4 as described above. After quenching the reaction, both samples were mixed in a 1:1 ratio according to protein amount (calculated by Pierce BCA Protein Assay Kit, Thermo Fisher Scientific) and the already mentioned lysis procedure was applied with the following changes: lysis was performed with 8 m urea without heating and supported by sonication (diagenode: Liège, Belgium Bioruptor, 3 × 30 s, 4 °C). Proteins were not precipitated. The label-swap experiment was repeated once, resulting in four replicates.

##### Protein Digestion and Enrichment of Cross-linked Peptides

Precipitated proteins were resuspended in 50 μl 8 m urea/50 mm ammonium bicarbonate, pH 8 (for the qualitative data set). Proteins were reduced and alkylated by adding DTT (10 mm, 1 h, RT) and iodoacetamide (IAA, 40 mm, 30 min, RT, in the dark), respectively, and were finally digested by trypsin (Promega) in an enzyme-to-protein ratio of 1:50 for 18 h at 37 °C. The digestion was terminated by adding TFA to a final concentration of 0.5%. SepPak cartridges (1cc, tC18, Waters, Milford, Massachusetts) were used for desalting according to manufacturer's instructions. After vacuum drying, peptide size exclusion chromatography (peptide SEC) was performed to enrich cross-linked peptides (59). Peptides were resuspended in peptide SEC running buffer (30% ACN/0.1% TFA). 300 μg were loaded onto a SuperdexPeptide 3.2/300 column (GE Healthcare, flow rate of 50 μl/min, 50 μl fraction volume). Fractions of column volume between 1.1 and 1.7 ml (1.5 ml for the quantitative data set) were vacuum dried, resuspended in loading buffer (2% ACN/0.05% TFA) and subjected to liquid chromatography tandem mass spectrometry (LC-MS/MS).

##### LC-MS/MS Analysis

Cross-linked peptides of every peptide SEC fraction subjected to LC-MS/MS were measured in technical duplicates on an Orbitrap Fusion or Fusion Lumos Tribrid Mass Spectrometer (Thermo Fisher Scientific), respectively. A Dionex UltiMate 3000 UHPLC system (Thermo Fisher Scientific) equipped with an in house-packed C18 column (ReproSil-Pur 120 C18-AQ, 1.9 μm pore size, 75 μm inner diameter, 30 cm length, Dr. Maisch GmbH, Ammerbuch, Germany) were coupled online to the mass spectrometer. Peptides were separated by applying a 180 min LC method. MS1 and MS2 spectra were acquired both in the orbitrap (OT) with a resolution of 120,000 and 30,000, respectively. In MS1, the scan range was set from 350 to 1550 *m/z*, AGC target to 5 × 10^5^ and maximum injection time to 60 ms. The dynamic exclusion time was set to 10 s, the 20 most abundant precursors with a charge state of 3–8 were selected for fragmentation per duty cycle. For MS2, higher-energy collisional dissociation (HCD) with a normalized collision energy of 30% was chosen for fragmentation. AGC target was set to 5 × 10^4^, injection time to 128 ms. The same settings were applied for the comparison of BS3 with DSS, but only one technical replicate was measured on Orbitrap Fusion Lumos.

For the quantitation, the following changes were applied: peptides were measured on a Q Exactive HF-X (Thermo Fisher Scientific). In MS1, AGC target was set to 1 × 10^6^, maximum injection time to 50 ms. Dynamic exclusion was set to 60 s and the 30 most abundant precursors were selected for fragmentation. For MS2, AGC target was set to 1 × 10^5^.

##### Data Analysis

All .raw files were converted into .mgf files with Proteome Discoverer 2.1 (Thermo Fisher Scientific, signal-to-noise ratio 1.5, 1000–10000 Da precursor mass). The software pLink 1 (60, 61) (v. 1.23, pFind group) was used for identification of cross-linked peptides. The following settings were applied: BS3 was used as cross-linker, BS3-d4 was added for quantitation analysis. Trypsin was set as digestion enzyme with maximum two missed cleavage sites. Carbamidomethylation on cysteine was defined as fixed modification, oxidation of methionine as variable modification. Precursor mass tolerance was set to ± 5 Da and adapted to ± 4 Da for the quantitation analysis. An additional filter tolerance of ± 10 ppm was applied around every isotopic peak. Fragment ion mass tolerance was set to 20 ppm. False discovery rate (FDR) of 1% at spectrum level was applied. A database with the 400 most abundant proteins (top400) for every biological replicate was generated by searching linear peptides with Mascot (62) (v. 2.3.02) and ranking proteins in Scaffold (63) (v. 4) by dividing total spectral counts by molecular weight. In Mascot the following settings were applied: peptide tolerance 10 ppm, fragment tolerance 0.02 Da, trypsin as protease with maximum four missed cleavage sites, carbamidomethylation on cysteine, oxidation of methionine and hydrolyzed BS3 (mass shift +156.077 Da) as well as Tris-quenched BS3 (mass shift +259.142 Da) as variable modification, 1% FDR. A reviewed yeast database (UniProt/SwissProt, 02/2016, 23481 entries) was used. Results were finally filtered for proteins originating from *Saccharomyces cerevisiae*. Because previous cross-linking studies (48, 64) showed that the majority of cross-linked proteins are within the most abundant ones and pLink 1 can perform a search against 400 proteins in a reasonable time, we chose this number to get reliable data. To not further increase the number of proteins we did not add contaminants for the cross-linked peptides search. Data sets were filtered by removing ambiguous identifications, cross-links only supported by a single cross-linked peptide spectrum match (CSM) and/or with a log_10_-transformed pLink 1 spectrum score below 4. By manual spectrum evaluation we observed that the spectrum score given by pLink 1 correlates with the spectrum quality. Spectra with a pLink 1 spectrum score below 4 are more likely to be not reliable.

For the comparison of BS3 with DSS and for the quantitation experiments, a label-swap-specific top400 protein database of a MaxQuant (65) (v. 1.6.0.1) search for linear peptides was provided. To generate such databases, MaxQuant was operated with default settings, except for two additional modifications: dead-end peptides with hydrolyzed BS3 and Tris-quenched BS3, respectively, and trypsin as protease with maximum three missed cleavage sites. The reviewed *Saccharomyces cerevisiae* database (UniProt/SwissProt, 12/2016, 6721 entries) was used. For ranking, iBAQ values were considered. Cross-linked peptides were quantified by XiQ (66) with default settings based on pLink 1 identifications (as described above). Heavy-to-light ratios on peptide level were calculated by dividing the sums of the respective areas under the curve of the first to third isotopic peak in .raw files by a drop to 10% signal intensity, the monoisotopic peak was ignored (these values were also taken to replace intensities given by pLink 1 because not all entries had valid values). Ratios were log_2_-transformed, median-normalized and collapsed to residue level with an in-house written R script based on Chen *et al.* (67). Briefly, median ratios were calculated for each charge state per peptide, which were then summarized to unique peptides as a weighted average based on the intensity of the peptide with a particular charge. Unique peptides were finally summarized to unique linked residue sites as median ratios of all supporting peptides.

##### Docking Experiments

Docking experiments have been performed using Rosetta software (68–70). The structure of the CIII_2_CIV_2_ mitochondrial respiratory supercomplex from *S. cerevisiae* (PDB: 6HU9 (71)) has been downloaded from the Protein Data Bank of Transmembrane Proteins (PDBTM (72)) providing transformed coordinates suited for docking experiments in the membrane bilayer. The required files describing membrane topology have been generated from the atomic models using tools available in Rosetta (mp_span_from_pdb, mp_dock_setup). Prior to docking experiments the docking partners have been manually preoriented in PyMOL based on spatial restraints derived from the cross-linking data. This approach avoided the necessity of using global and low-resolution docking steps. Hence, all docking experiments have been performed using the local docking approach (high resolution docking) with spatial constraints obtained from cross-linking experiments. These constraints have been applied in the form of “AtomPair” harmonic distances (9.0 Å ± 2.5 Å) between the cross-linked atoms (NZ of lysine residues and N of the N-terminal residue). For each docking experiment at least 5000 decoys have been generated. These decoys have been ranked based on the interface score (I_sc) which represents the energy of the interactions across the interface and subsequently clustered.

The atomic models of docked proteins Ndi1 and Pet9 have been obtained from the deposited coordinates of type-II mitochondrial NADH dehydrogenase (PDB: 4G73 (73)) and yeast mitochondrial ADP/ATP carrier protein (PDB: 4C9G (74)), respectively. The atomic model of Min8 has been calculated with Rosetta using *ab initio* protocol designated for *de novo* folding of membrane proteins (75, 76). The topology of transmembrane region and fragment files have been generated using the OCTOPUS server (77) and the Robetta server (robetta.bakerlab.org), respectively. Over 300,000 low-resolution centroid models have been generated of which the 5000 most energetically favored have been selected for clustering and subsequently converted to “full-atom” models. The orientations of intermembrane space and outer membrane domains of the docked Min8 model have been manually adjusted using Coot (78).

##### Visualization of Cross-linking Results

Cytoscape (79) and xVis (80) were used for visualizing protein-protein interactions. Perseus (81) was used for illustrating the volcano plot. Structures were represented by PyMOL (www.pymol.org, Schrödinger LLC) and UCSF Chimera (82). Accessible interaction space was calculated by DisVis (83).

##### Miscellaneous

Standard techniques were used for SDS-PAGE and Western blotting on polyvinylidene fluoride (PVDF) membranes. BN-PAGE were performed by solubilization of mitochondria in solubilization buffer (20 mm Tris-HCl pH 7.4, 5 mm EDTA, 50 mm NaCl, 10% glycerol and 1 mm PMSF supplemented with either 1% digitonin or 0.6% DDM) for 20 min at 4 °C. The lysate was cleared at 20,000 × *g* at 4 °C for 10 min. The supernatants were mixed with 10x loading dye (5% Coomassie G-250, 500 mm 6-amino-hexanoic acid and 0.1 m Bis-Tris, pH 7.0) and separated on a 4–10% or 6–10% polyacrylamide gradient gel followed by Western blotting.

##### Experimental Design and Statistical Rationale

**Fig. 1.**
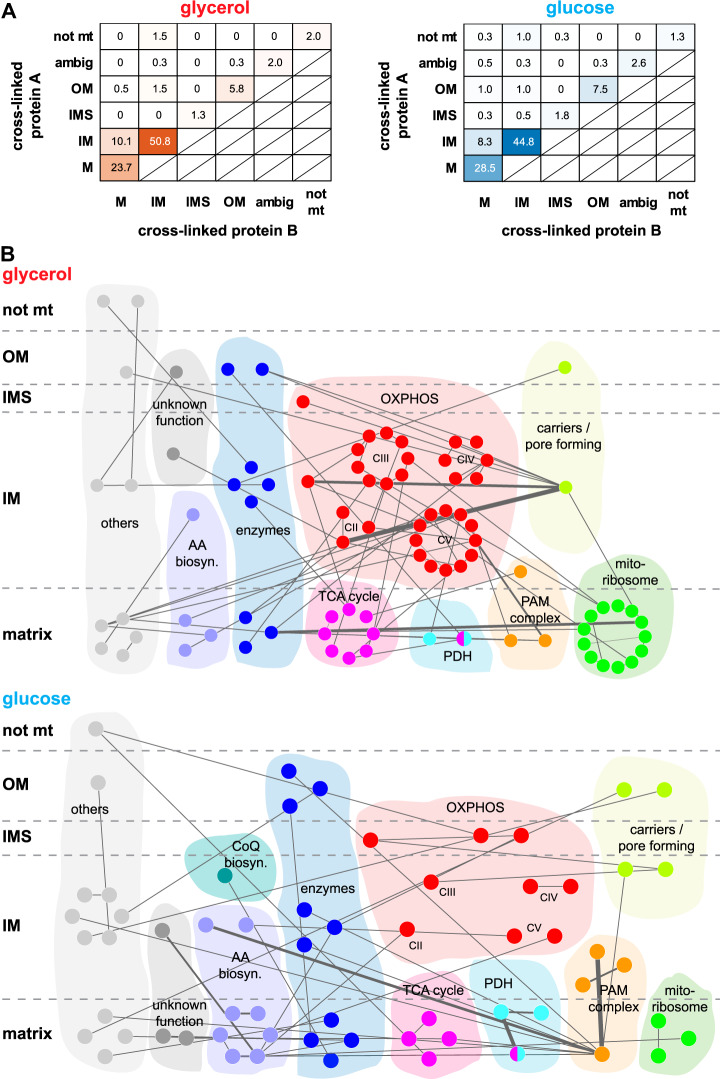
**Suborganellar protein localization and global protein-protein interaction network.** *A*, Relative distribution of proteins within the different compartments of yeast mitochondria purified from yeast grown on glycerol (red, left) or glucose (blue, right) medium in percent. Localization of protein A is plotted against the location of its cross-linked partner protein B. Significance of differences between both conditions were calculated by a Chi-square test of independence with an assumed alpha level of 0.05. All combinations, X^2^ = 0.043; combinations IM-IM, M-M, IM-M, X^2^ = 0.008. *B*, Protein interaction networks for glycerol (upper panel) and glucose (lower panel). Only those interprotein connections are displayed that were uniquely identified in the respective conditions but whose proteins were identified in both conditions. Localizations based on Vögtle *et al.* (11). Thickness of the edges is proportional to the number of unique residue pairs. OM, outer membrane; IMS, intermembrane space; IM, inner membrane; M, matrix; ambig, ambiguous; not mt, not mitochondrial; AA biosyn., amino acid biosynthesis; CoQ biosyn, Coenzyme Q biosynthesis; OXPHOS, oxidative phosphorylation system; TCA, tricarboxylic acid; PDH, pyruvate dehydrogenase complex; PAM, presequence translocase-associated motor; mitoribosome: mitochondrial ribosome. Networks visualized by Cytoscape (79).

In total, 78 .raw files were analyzed within the qualitative data set and 62 .raw files within the quantitative data set. For the qualitative data set, purified mitochondria derived from two different yeast cell batches per condition were used for the cross-linking experiments. After peptide SEC, each fraction was analyzed twice by LC-MS/MS, resulting in a total of two biological replicates per condition and two technical replicates per fraction. For the quantitative data set, label-swap cross-linking experiments were performed on purified mitochondria derived from two different cell batches per condition, resulting in two biological replicates split into four labeled replicates (one “forward” and one “reverse” sample per biological replicate). Each peptide SEC fraction was analyzed twice by LC-MS/MS. To determine if the results of the quantitation experiments were significant a one-sample *t* test was applied to the median normalized data, taking a minimum of three valid values out of four replicates into account. Differences with a *p* value ≤ 0.05 and a fold change ≥ 2 were considered as significant. For the comparison of BS3 *versus* DSS, one biological replicate (crude mitochondrial extract from yeast grown on glycerol medium) was considered and every peptide SEC fraction was analyzed once by LC-MS/MS. This approach provided appropriate results to judge that BS3 is also suitable for cross-linking in purified organelles. Significance of differences between the cross-linked protein pairs identified in both conditions (represented in Fig. 1*A*) were calculated with a Chi-square test of independence. For that, absolute numbers of unique protein-protein interactions (including both intra- and interprotein cross-links) were considered. All “ambig”- and “not mt”-pairs were combined, OM-M-pairs were summed up with OM-IM-pairs and both IMS-M-pairs and IMS-IM-pairs were summed up with IMS-IMS-pairs to keep the number of values per category ≥ 5, suitable to perform a robust Chi-square test of independence. Values for X^2^ below an alpha level of 0.05 were considered as significant.

## RESULTS

### 

#### 

##### BS3 Is A Suitable Cross-linker for Proteins in All Mitochondrial Subcompartments

To determine if the water-soluble but membrane-impermeable cross-linker BS3 is an appropriate protein-protein cross-linker for organelles, we purified mitochondria from yeast cells grown on medium containing glycerol as a carbon source (58). Mitochondria that are purified under these conditions remain import competent for *in vitro* transport studies and possess a coupled oxidative phosphorylation system indicative of a tight inner membrane (84–86). Purified mitochondria that were used for cross-linking analyses were incubated with an equimolar mixture of water-insoluble but membrane-permeable DSS and isotopically labeled BS3-d4. After the reaction, mitochondria were lysed, proteins digested, and cross-linked peptides enriched by peptide size exclusion chromatography (peptide SEC), peptide SEC fractions containing cross-links were analyzed by LC-MS/MS and searched against a dedicated database with pLink 1 (60, 61). We quantified the obtained cross-links by XiQ (66) and determined the suborganellar location of the cross-linked proteins. Although the majority of quantified peptide residue pairs remained unchanged, we found that ∼72% of cross-linked proteins with quantified residue pairs showing a fold change ≥ 2 belonged either to the inner membrane (IM) or to the matrix, regardless of the cross-linker (supplemental Fig. S1, supplemental Table S1). This showed, that despite integrity of mitochondrial membrane, BS3 reacts with proteins of all mitochondrial subcompartments. Because BS3 seems to be more suitable for cross-linking organelles in aqueous buffer conditions as compared with DSS, which needs organic solvents for resuspension, we used BS3 for our further studies.

##### Carbon Source Specific Cross-linking of Mitochondria

Yeast cells are able to grow under fermentative and non-fermentative conditions. Although fermentative growth largely bypasses mitochondrial functions, under non-fermentative conditions oxidative phosphorylation becomes essential. To assess mitochondrial protein networking under these two metabolic conditions, we grew yeast on media containing glucose or glycerol as a carbon source. Mitochondria were isolated, cross-linked with BS3, and analyzed regarding the differences in protein-protein interactions. Cross-linked peptides were enriched and analyzed as described above. To search the cross-linked peptides against a database, we first generated a replicate-specific protein database containing the 400 most abundant proteins that have been identified through a canonical search of the linear (*i.e.* non-cross-linked) peptides against the proteome of *S. cerevisiae* (supplemental Table S2). The dedicated databases were used for the cross-linked peptides search with pLink 1 of two biological replicates for each set of growth condition and two technical replicates for each biological replicate. The search revealed 2999 and 2595 unique residue pairs under glycerol and glucose condition, respectively, with a false discovery rate (FDR) of 1% at spectrum level. For further analysis, we excluded those residue pairs that were only identified by a single CSM and had a pLink 1 spectrum score of < 4. Thus, the overall number of unique residue pairs was reduced to 2100 and 1787 under glycerol and glucose condition, respectively. 17% (359) and 15% (266) of these were cross-links between two different proteins (supplemental Table S3). The filtering also increased the overlap of unique residue pairs between biological replicates from 39% to 55% in the glycerol condition and from 45% to 62% in the glucose condition (supplemental Fig. S2). To this end, we generated a global interaction map of cross-linked mitochondrial proteins derived from yeast grown on glycerol or glucose to define differences in the detected protein-protein interactions (Fig. 1). The data revealed a total of 261 (glycerol) and 260 (glucose) cross-linked proteins with an overlap of 66%. These accounted for 396 and 386 unique protein-protein interactions, respectively, split into 42% (167) interprotein links and 58% (229) intraprotein links in the glycerol condition and 40% (155) interprotein links and 60% (231) intraprotein links in the glucose condition. For comparison of the different growth conditions we plotted the suborganellar locations of these proteins (Fig. 1*A*), based on the study by Vögtle *et al.* (11). Most cross-links in mitochondrial proteins from both growth conditions were found between inner membrane (IM) proteins, followed by mitochondrial matrix (M) protein interactions, whereas cross-links within and between outer membrane (OM) proteins were less abundant. Protein cross-links that span entire membranes - *e.g.* cross-links between OM and/or intermembrane space (IMS) proteins and matrix proteins - are very unlikely to occur and consequently represent less than 5% of all cross-links in both data sets.

Interactions between inner membrane proteins (IM-IM) were more abundant in mitochondria from glycerol medium compared with mitochondria isolated from cells grown on glucose medium (201 (51%) *versus* 173 (45%)). In contrast, interactions between matrix proteins (M-M) were more frequent under glucose condition (94 (24%) *versus* 110 (29%). Cross-links of outer membrane proteins were almost equally represented (32 (8%) and 38 (10%)) in mitochondria from both growth conditions. As calculated by a Chi-square test, the observed differences between cross-linked protein pairs in both conditions are significant. This effect is even more distinctive for the densely protein packed inner membrane and the matrix of mitochondria (combinations IM-IM, M-M, IM-M, Fig. 1*A*).

We then grouped proteins involved in interprotein cross-links and the respective protein complexes according to their function and localization (Fig. 1*B* and supplemental Fig. S3). Seventy-four interprotein cross-links among the complexes of the oxidative phosphorylation system (OXPHOS), the tricarboxylic acid (TCA) cycle, and the mitochondrial ribosome were common to both data sets. Also, subunits of the presequence translocase-associated import motor (PAM) complex, the pyruvate dehydrogenase complex (PDH), enzymes such as transferases, isomerases, desulfurases and oxidases (*e.g.* Cpr3, Isd11 and Alo1), proteins involved in amino acid biosynthesis (*e.g.* Ilv2, Ilv5, Ilv6) as well as carriers and pore forming proteins (*e.g.* Pet9, Por1, Tom40) were identified under both growth conditions (supplemental Fig. S3). Other cross-linked proteins that do not fall into any of these categories are listed as “others”, *e.g.* Prohibitins (Phb1, Phb2), DNA-binding proteins (*e.g.* Abf2), and translational activators (*e.g.* Mss51). In addition, we identified cross-links between proteins with unknown function according to the SGD database, *e.g.* Om45, Fmp40, and Aim17.

Differences in the protein interactions based on our cross-linking data were found in case of proteins of the OXPHOS system, the TCA cycle, proteins involved in amino acid biosynthesis, the mitochondrial ribosome, the PAM complex and the PDH (Fig. 1*B*). More interprotein cross-links were identified between proteins involved in oxidative phosphorylation, metabolic pathways, and the TCA cycle in mitochondria from glycerol medium. In contrast, interprotein cross-links of proteins involved in 2-oxocarboxylic-acid metabolism and the biosynthesis of secondary metabolites and amino acids were found to be more abundant under glucose condition, whose copy numbers of proteins involved in the OXPHOS system or the TCA cycle are reduced because of glucose-repression of the respective genes (56, 57).

##### Different Interprotein Cross-links Among IM Proteins Under Different Growth Conditions

**Fig. 2.**
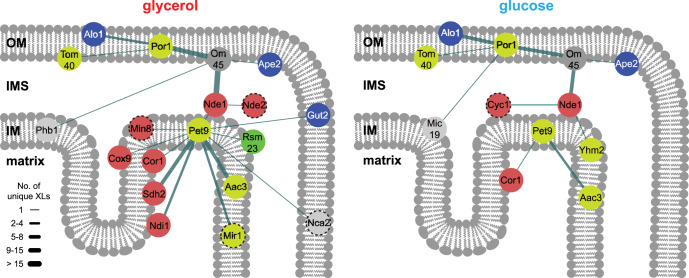
**Interaction networks of outer and inner membrane proteins.** Displayed are proteins with a high number of unique residue-to-residue cross-links, like Por1, Om45, Nde1 and Pet9, and their interactions to other mitochondrial proteins either under glycerol (left) or glucose (right) condition. Proteins are colored according to their category depicted in Fig. 1 (red, ETC; blue, amino acid biosynthesis; yellow, carriers and pore forming proteins; green, mitochondrial ribosome; light gray, others; dark gray, unknown function). Dashed circles: Protein was not part of the database of the other condition. Thickness of the edges is proportional to the number of unique residue pairs. OM, outer membrane; IMS, intermembrane space; IM, inner membrane.

We analyzed in detail the changes of protein interactions among outer and inner membrane proteins. For this, we focused on two abundant OM proteins, Por1 and Om45 and their interactions with other proteins (Fig. 2). Under glycerol and glucose condition, we identified eleven and eight unique residue-to-residue cross-links, respectively, between Om45, a major outer membrane constituent of so far unknown function (87, 88), and Por1, for which interactions had been described previously (89). Por1 is a voltage-dependent anion channel and connects the IMS with the cytosol. Cross-links of Por1 to Alo1, the d-arabinono-1,4-lactone oxidase, and to Tom40, the channel forming unit of the translocase of outer membrane (TOM) complex, are also observed under both conditions. An interaction between Por1 and Alo1 has not yet been described. Additionally, we identified a very high number of unique residue-to-residue cross-links between Om45 and Nde1, the external NADH:ubiquinone oxidoreductase, suggesting a large interaction region between these proteins. An interaction between these proteins has also not been described yet.

We also observed different interaction patterns of IM proteins depending on the yeast's growth condition. One of the most abundant proteins in the inner membrane is Pet9, the major ADP/ATP carrier (90). Pet9 cross-linked to eleven other proteins in mitochondria purified from glycerol grown cells, but only to two proteins under glucose condition (Fig. 2). Among these eleven proteins, three proteins belong to the ETC, namely Sdh2 (CII), Cor1 (CIII) and Cox9 (CIV), indicating an association of Pet9 to the ETC, which is consistent with previous results (91, 92). Pet9 also cross-linked to Ndi1 and Min8; the latter is an 8 kDa protein of unknown function, whose possible function and localization are discussed below. Under glucose, Pet9 also cross-linked to Cor1 and additionally to its paralogue Aac3. These results indicate that the detection of interaction patterns of inner membrane proteins, especially of Pet9 and ETC proteins, depend more on the growth conditions than those among outer membrane proteins (*e.g.* Por1 and Om45). This is based on higher copy numbers of proteins belonging to the ETC and Pet9 under non-fermentative growth conditions (55) because of glucose-repression (56, 57).

##### Quantitative Cross-linking in Dependence On the Carbon Source and Protein Abundances

**Fig. 3.**
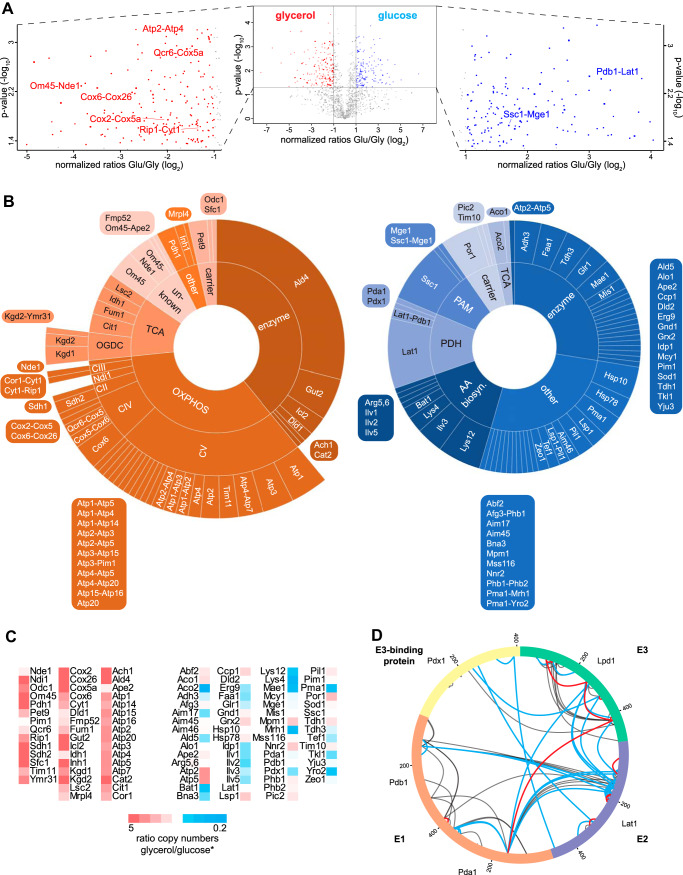
**Quantitative cross-linking analysis of mitochondria isolated from yeast grown on glycerol- or glucose-containing medium.** *A*, Volcano plot representing quantified cross-linked residue peptide pairs. Negative log_10_-transformed *p* value is plotted against normalized log_2_-transformed heavy (glucose, blue) to light (glycerol, red) ratios. Residue pairs with a *p* value ≤ 0.05 and a fold change ≥ 2 were considered as significantly changed. Left and right boxes, zoomed area for glycerol and glucose condition, respectively, with exemplary labeled interprotein cross-links of different quantities. *B*, Protein interactions clustered according to their category described in Fig. 1, depicted as sunburst diagrams. Cross-links with higher intensity under glycerol condition are shown in the left diagram (red) and under glucose condition in the right one (blue). Area of the fields is proportional to the number of unique residue pairs. *C*, Ratios of copy numbers of each quantified protein in both of the conditions. Red, higher copy number in glycerol; blue, higher copy number in glucose. *, values taken from Morgenstern *et al.* (55). *D*, Intraprotein- and interprotein cross-links within the pyruvate dehydrogenase complex (PDH) identified in the qualitative data sets. Blue lines, cross-links only identified under glucose condition; red lines, cross-links only identified under glycerol condition; black lines, common links. Visualized by xVis (80).

The observed differences in the cross-linking patterns of mitochondrial proteins purified from glycerol or glucose media might be because of different abundance levels of proteins under the different growth conditions or to alterations in protein interactions including the quaternary arrangement of proteins in complexes. To investigate this more closely we performed quantitative protein cross-linking under both growth conditions using isotopically labeled cross-linkers. Mitochondria isolated from yeast grown on glycerol were cross-linked with BS3-d4 whereas mitochondria from glucose-grown yeast were incubated with BS3 and vice versa. Cross-link identification was performed by pLink 1 and quantification by XiQ on MS1 level (supplemental Table S1). Data analysis was performed on ratios obtained in three of four replicates with an abundance fold change greater than two and a *p* value below 0.05 considered as significant. In total, 169 cross-links (intra- and interprotein), accounting for 46 proteins, showed significantly higher MS1 intensities under glycerol condition. Under glucose condition, 145 intra- and interprotein cross-links, representing 62 different proteins, had significantly higher MS1 intensities (Fig. 3*A*). Of the 169 cross-links derived from the glycerol data set, 134 (79%) were intraprotein cross-links, and from the glucose data set 135 (93%). Enrichment analysis of the significantly different cross-linked proteins using the KEGG database (93) confirmed that more proteins of the oxidative phosphorylation system, general metabolism and the TCA cycle are cross-linked under glycerol condition. Proteins belonging to the 2-oxocarboxylic acid metabolism, biosynthesis of secondary metabolites and amino acids are cross-linked in higher yield under glucose condition. This reflects the strong influence of glucose-repressed genes encoding for proteins belonging to the OXPHOS system (56, 57). The diagrams in Fig. 3*B* illustrate the significantly more abundant cross-linked proteins under either condition according to their assignment for the qualitative data set (Fig. 1*B*). Cross-links of Ilv2, Ilv3, Ilv5, Lys4 and Lys12 of the amino acid biosynthesis pathway show increased intensities under glucose condition. Also, intra- and interprotein cross-links *e.g.* between Ssc1 and Mge1 showed increased intensity under glucose condition. Furthermore, intraprotein cross-links of the OM transporter Por1 were more abundant under glucose condition (Fig. 3*B*, right diagram).

Under glycerol condition, Ald4 showed the highest number of unique cross-links. Similarly, intraprotein cross-links of CII-CV and interprotein cross-links between Qcr6 of CIII and Cox5a of CIV have higher intensities. Moreover, the intensities of cross-linked peptides between Om45 and Nde1 were more abundant under glycerol condition (Fig. 3*B*, left diagram). This quantitative analysis of cross-links supported our qualitative results described above.

However, most of the quantified cross-links do not reflect conformational changes of single proteins under the different conditions. Instead, the quantitative cross-linking results mirror the different abundances of proteins in mitochondria grown under glycerol *versus* glucose condition. The majority of proteins that show higher cross-linking abundances under glycerol condition are also present in higher copy numbers (55). Fig. 3*C* lists the proteins that show significantly higher intensities of cross-linked peptides under either condition and relates these proteins to their copy numbers. In this regard, all quantified intraprotein cross-links are presumably because of higher copy numbers of the respective proteins as induced by the respective carbon source. Nevertheless, quantitative changes in interprotein cross-links can indeed provide valuable insights to changes of interactions between equally expressed proteins; this is exemplified by the pyruvate dehydrogenase complex.

##### Quantitative cross-linking Defines Organization of Pyruvate Dehydrogenase Complex

We evaluated the quantitative data set for those proteins that have equal copy numbers under both glycerol and glucose conditions (according to (55)) but showed different cross-linking yields. We identified proteins of the pyruvate dehydrogenase complex (PDH), a multienzyme complex located in the matrix of mitochondria. It converts pyruvate into acetyl-CoA and, hence, connects glycolysis with the TCA cycle. E1, the pyruvate dehydrogenase, consists of two subunits, Pda1 and Pdb1, and - together with E3, the dihydrolipoyl dehydrogenase Lpd1 - it surrounds E2, the dihydrolipoyl transacetylase Lat1. The assembly of E2 and E3 is supported by the E3-binding protein, Pdx1, forming a fully assembled PDH (94). Lpd1 shows a 2.4-fold higher copy number under glycerol condition and Pdx1 a 1.4-fold higher copy number under glucose condition (55). Pda1, Pdb1 and Lat1 are equally expressed under both growth conditions. Under glucose condition 13 unique intraprotein cross-links of Lat1, one intraprotein cross-link in each of the proteins Pda1 and Pdx1, and two interprotein cross-links between Lat1 and Pdb1 are more abundant. These quantitative cross-linking results reflect structural arrangements of equally expressed protein components in organelles under different conditions.

Unfortunately, the significance of the quantitative analysis does not allow for a more detailed assessment of the assembly state of the PDH dependent on the carbon source. We thus took our qualitative study, *i.e.* the separate XL-MS analyses of mitochondria isolated from yeast grown under two different conditions into account. Here, the number of unique residue pairs within the PDH was also higher under glucose condition with 40 cross-links identified under glucose and only 14 under glycerol condition (supplemental Table S3). Strikingly, the E3-binding protein Pdx1 forms two unique residue-to-residue cross-links under glucose condition with each of E2 and E3, but only one unique residue-to-residue cross-link was identified under glycerol condition between Pdx1 and E3 and no cross-link to E2. Interprotein cross-links between E2 and E3 were only identified under glucose condition (Fig. 3*D*). Both the quantitative and qualitative XL-MS data suggest that fully assembled PDHs - *i.e.* in which Pdx1 brings E3 into proximity with E2 - are presumably more present under glucose condition.

##### Cross-links Relate Ndi1 to ETC Supercomplexes

A main function of mitochondria is the production of energy stored in the form of ATP. For this, the ETC, which consists of three highly abundant multi-subunit complexes in yeast (CII-CIV), generates an electrochemical gradient across the inner membrane that drives ATP production by the F_1_F_o_ ATP synthase (complex V, CV). Unlike mammals, *S. cerevisiae* does not possess a complex I. Instead, it utilizes Ndi1, a NADH:ubiquinone oxidoreductase on the matrix side that does not pump protons (95).

**Fig. 4.**
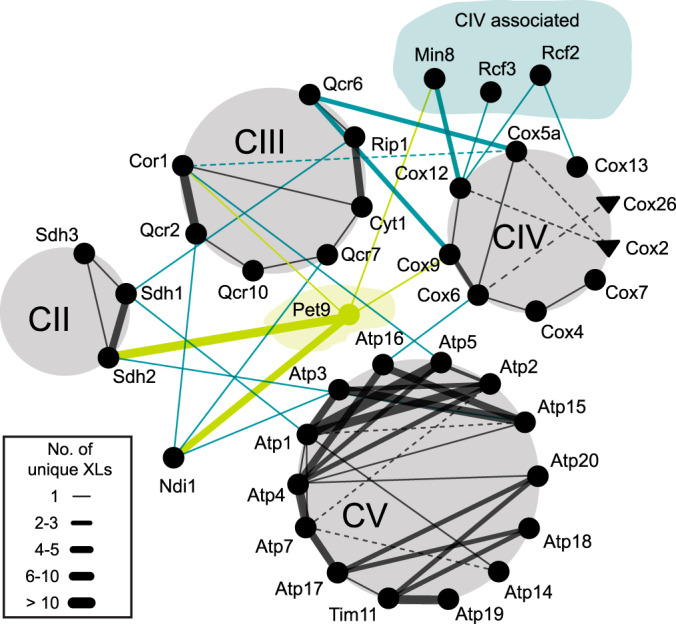
**Interaction network within the OXPHOS system identified in the glycerol data set.** Ndi1, CIV associated proteins and Pet9 are included. Only interprotein cross-links are displayed. Solid and dashed lines indicate interactions identified in the qualitative and the quantitative data set, respectively. Circles and triangles indicate proteins identified in the qualitative and quantitative data set, respectively. Thickness of the edges is proportional to the number of unique residue pairs. Black lines, intra-complex cross-links; turquoise lines, inter(-complex) cross-links.

The presence of CIII-CIV supercomplexes and the association of CII has been described in yeast (18, 96). Whether Ndi1 is directly associated with one of the sub- or supercomplexes has not been described yet. In the following we exclusively consider cross-links identified in mitochondria isolated from yeast grown on glycerol-containing medium, because here genes encoding proteins of the OXPHOS system are not glucose-repressed. Cross-links show that proteins of the single OXPHOS complexes are connected including Ndi1, which forms cross-links to CIII and CV (Fig. 4). Several unique inter-complex cross-links were identified between Qcr6, a subunit of CIII, and Cox5a and Cox9, both subunits of CIV. In the structure of the CIII-CIV supercomplex, Qcr6 and Cox5a are near the interface of CIII and CIV (97). The location of the cross-links in purified organelles agrees with the recently published structure of a yeast CIII_2_CIV_2_ supercomplex (71). Remarkably, we found that Ndi1 cross-links to proteins of CIII, with residues K76 of Ndi1 and K17 of Qcr7, and K361 of Ndi1 and K198 of Qcr2. A potential interaction space of Ndi1 and CIII_2_CIV_2_ supercomplex was calculated by DisVis (83) by using the identified cross-linking sites and the structures of both the CIII_2_CIV_2_ supercomplex and Ndi1 (73), indicating no steric hindrance between Ndi1 and CIII or CIV (supplemental Fig. S4*A*).

**Fig. 5.**
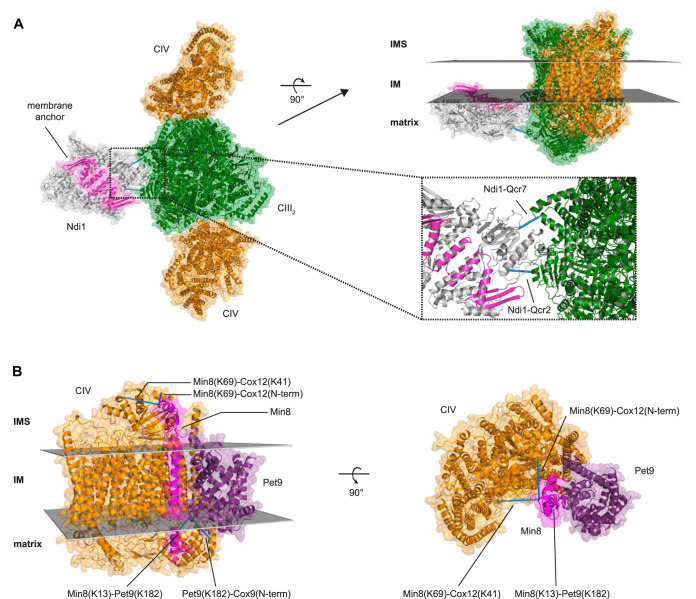
**Structural validation of supercomplexes.** *A*, Representation of a Ndi1CIII_2_CIV_2_ supercomplex as top view from IMS and as side view embedded into IM. Ndi1 dimer (gray; light purple, membrane anchor; PDB: 4G73 (73)) was docked to the CIII_2_CIV_2_ supercomplex structure (green, CIII dimer; orange, CIV monomers; PDB: 6HU9 (71)). Inlet, zoomed interaction interface between CIII and Ndi1. *B*, *Ab initio* modeled Min8 (magenta) and Pet9 (purple; PDB: 4C9G (74)) docked to cytochrome *c* oxidase (extracted from PDB: 6HU9 (71)) as top view from IMS and as side view embedded into IM. Identified cross-links are represented as blue bars, satisfying the distance constraint of 30 Å given by the cross-linker BS3. IMS, intermembrane space; IM, inner membrane.

For a detailed quaternary arrangement of an Ndi1CIII_2_CIV_2_ supercomplex, we docked the dimeric structure of Ndi1 to the CIII_2_CIV_2_ supercomplex structure by Rosetta (68–70, 72). As restraints we used the cross-links to CIII and the location of the C-terminal membrane anchor of Ndi1 (Fig. 5*A*). The arrangement of Ndi1 and the CIII_2_CIV_2_ supercomplex structure is comparable to that of the mammalian CICIII_2_CIV supercomplex (supplemental Fig. S4*B*). A Ndi1_2_CIII_2_CIV_2_ supercomplex is also conceivable by docking Ndi1 on both sides of the dimeric CIII, forming a propeller-like structure with Ndi1-CIII_2_-Ndi1 on the one axis and CIV-CIII_2_-CIV on the other axis. The cross-links reveal that Ndi1 is indeed a part of an ETC supercomplex.

##### Min8 is Associated with the Cytochrome c Oxidase

In addition to Ndi1, we found that Pet9, Rcf2, Rcf3, and Min8 cross-linked to ETC complexes in the glycerol data set (Fig. 4). Rcf proteins have been described to interact with CIV and Rcf1 is involved in formation of supercomplexes CIII-CIV (86, 98, 99). Recently, Rcf3 was characterized as homolog of Rcf2, both of which associate with CIII and CIV (98). In our data set, the N terminus of Rcf3 cross-linked to the N terminus of Cox12, a subunit of CIV, whereas Rcf2 cross-linked through its C-terminal region to both Cox12 and Cox13, consistent with a proposed orientation of both Rcf2 and Rcf3 in the IM (98). Min8 is an uncharacterized protein that consists of 72 amino acid residues. Its C-terminal region cross-linked to Cox12 at the same amino acid position as Rcf2 and Rcf3. Additionally, the N-terminal region of Min8 cross-linked to Pet9 (Fig. 5*B*). Min8 has been already identified as a protein of the IM (55), with its C terminus facing the IMS, its N terminus facing the matrix, and a transmembrane region between amino acids 20 and 40. We calculated an atomic model of Min8 *ab initio* by Rosetta (75–78). This model was docked to CIV according to the identified cross-links, using the available CIV structure within the recently published CIII_2_CIV_2_ supercomplex (71). Additionally, Pet9 was integrated in the docking calculations based on the cross-links to Min8 and CIV. Fig. 5*B* shows the structure of CIV in which we have docked the modeled Min8 structure with its helical transmembrane domain located in close proximity to the helical transmembrane domains of the proteins of CIV. The C terminus of Min8 faces Cox12 on the IMS side and the N terminus of Min8 faces Pet9 on the matrix side. Cross-linked lysine residues of Min8 to Cox12 and to Pet9 meet the distance restraints of the cross-linker BS3. These results point toward the existence of a supercomplex, when one considers that Pet9, which is cross-linked to Min8, also forms interprotein cross-links to CII, CIII, and Ndi1 (Fig. 4).

**Fig. 6.**
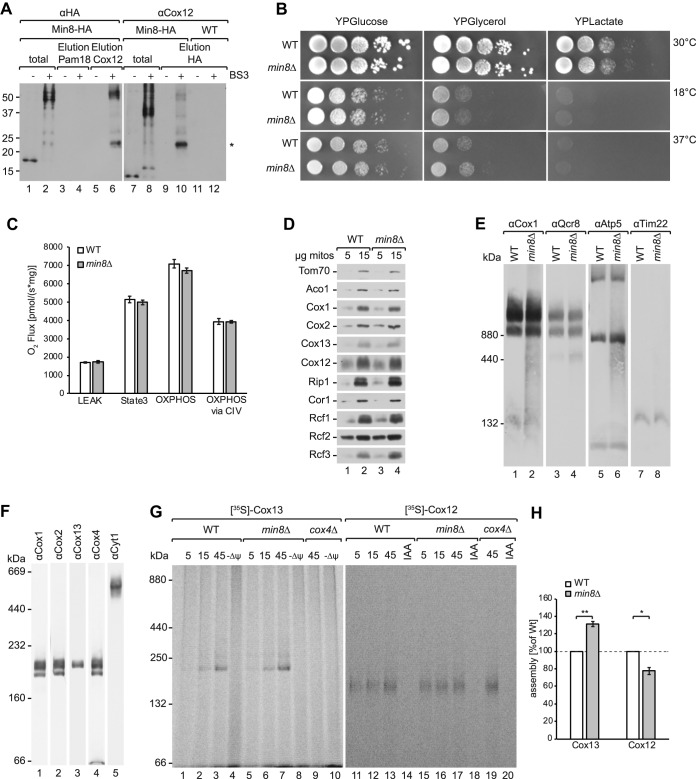
**Deletion of *MIN8* acts on Cox12 assembly.** *A*, Wild type (WT) and Min8-HA mitochondria were treated with BS3 cross-linker, solubilized with 0.5% Triton X-100 and 0.1% SDS containing buffer and co-immunoprecipitated with Cox12 and HA-antibodies. Total and eluate samples were analyzed by SDS-PAGE and Western blotting. The 22 kDa bands (marked with an asterisk) in lanes 6 and 10 represent the interaction between Min8 and Cox12, induced by the cross-linker BS3. Lanes marked with a “-“ represent the control experiments without prior cross-linking. *B*, Growth test of WT and *min8*Δ yeast cells on YPD, YPG and YPL media at 18 °C, 30 °C and 37 °C. *C*, Oxygen consumption measurements of mitochondria from WT and *min8*Δ (*n* = 3) (LEAK = non-phosphorylating respiration; State3 = saturating conditions; OXPHOS = maximal capacity for oxidative phosphorylation under coupled conditions; OXPHOS via CIV = complex IV capacity addressed by inhibition of electron transfer between CIII and CIV). Error bars indicate mean ± s.e.m. *D*, Isolated WT and *min8*Δ mitochondria were subjected to SDS-PAGE and analyzed by Western blotting. *E*, Mitochondria isolated from WT and *min8*Δ strains were solubilized in 1% digitonin buffer, subjected to BN-PAGE and Western blotting. *F*, WT mitochondria were solubilized in 0.6% DDM buffer and analyzed by BN-PAGE and Western blotting. *G*, Radiolabeled Cox13 and Cox12 were imported into WT, *min8*Δ and *cox4*Δ mitochondria for indicated times and treated with Proteinase K. Samples were lysed in 0.6% DDM buffer, analyzed by BN-PAGE and digital autoradiography. *H*, Quantification of three individual Cox13 and Cox12 assembly experiments in *min8*Δ compared with WT mitochondria (100%). A two-tailed unpaired *t* test was performed to assess statistical significance (*, *p* < 0.05; **, *p* < 0.005). Error bars indicate mean ± s.e.m.

The finding that Min8 associated with CIV suggests that it could represent a novel structural subunit of the complex. To further support this hypothesis, we performed cross-linking analyses and subsequently immunoisolated Min8 or Cox12 under denaturing conditions (Fig. 6*A*). These analyses supported a direct interaction between Min8 and Cox12. To address the functional relevance of Min8 association to CIV, we generated a *min8*Δ strain and tested these cells for growth on fermentable and non-fermentable media. *min8*Δ cells did not display a growth difference compared with the wild type under any of the tested conditions (Fig. 6*B*). Also, the steady protein levels of complex IV subunits were not significantly different between wild type and *min8*Δ (Fig. 6*D*). In agreement with this, activity and amount of CIV were not affected in the absence of Min8 (Fig. 6*C*, 6*E*). Previous studies showed that CIV displays heterogeneity in its constituents when analyzed by BN-PAGE after DDM solubilization, which dissociates supercomplexes. Core subunits of CIV such as Cox1, Cox2, and Cox4 are present in three different complexes, whereas Cox13 is only present in one specific form of CIV (86) (Fig. 6*F*). Cox12 and Cox13 are positioned at the periphery of the complex and in the assembly process of CIV integrated at a late stage. Considering the observed cross-links between Min8 and Cox12, we analyzed if the absence of Min8 affected the biogenesis of Cox12. For this, we imported radiolabeled Cox12 and Cox13 into purified wild type and *min8*Δ mitochondria. As a control, we imported both proteins into *cox4*Δ mitochondria, which lack mature CIV. After import, mitochondria were solubilized in DDM and protein complexes were separated by BN-PAGE. These analyses showed that imported Cox13 assembled into CIV in a Min8 independent manner. The assembly of Cox13 was even slightly but reproducibly increased in the absence of Min8 (Fig. 6*G*, 6*H*). In contrast, assembly of Cox12 occurred slower in *min8*Δ mitochondria (Fig. 6*G*, 6*H*). Interestingly, the observed Cox12-containing complex was apparent in *cox4*Δ mitochondria. Accordingly, the Cox12 complex does not represent mature CIV but rather an undefined new assembly intermediate of Cox12. We concluded, that Min8 promotes assembly of Cox12 into an intermediate complex whereas it appears to negatively affect the integration of Cox13 into CIV.

## DISCUSSION

In this study we used XL-MS to investigate the mitochondrial interactome of *Saccharomyces cerevisiae* grown on either fermentable (glucose) or non-fermentable (glycerol) carbon sources. Beside a qualitative approach, we also followed a quantitative one by using an isotopically labeled cross-linker. We first demonstrated that BS3 as water-soluble, but membrane-impermeable cross-linker is equally suited to cross-link proteins within all mitochondrial subcompartments compared with the water-insoluble but membrane-permeable cross-linker DSS. Although a slight disruption of the membranes because of the treatment with the cross-linker cannot be negated in either case, the use of BS3 avoids any organic solvents that might interfere with the membrane systems (100). Furthermore, our results indicate that a non-cleavable cross-linker is suitable to interrogate protein-protein interactions in more complex systems. This is in line with the conclusions drawn by Ryl *et al.* (49) who used DSS in their XL-MS study on human mitochondria.

We identified 2100 and 1787 unique residue pairs under glycerol and glucose conditions, respectively, with an FDR of 1% on spectral level. FDR values on peptide or protein level will be higher, whose practicability is currently discussed within the cross-linking community (101). We have focused on only the 400 most abundant proteins as identified from a linear search of non-cross-linked peptides and applied stringent filtering criteria to achieve reliable results on protein-protein interactions. Hence, our XL-MS results provide less protein interactions in terms of numbers when compared with similar studies performed in mammalian mitochondria (47–49). When we lower our criteria to 1 CSM at 1% FDR (supplemental Table S3) we reach very similar numbers of cross-links like those studies that investigated rodent mitochondria (47, 48). While our manuscript was in preparation, Makepeace *et al.* (50) published their method-driven XL-MS study of yeast mitochondria describing a workflow with the enrichable, MS-cleavable and isotopically labeled cross-linker CBDPS. Their data set covers 76% of the protein-protein interactions in our study. The overlap of the data from very similar samples from two different laboratories demonstrates the high specificity and reproducibility of the XL-MS method *per se*, despite the use of different experimental settings in terms of cross-linker, sample fractionation and computational data analysis. Hence, it can be expected that XL-MS applied to cellular systems will become a promising tool in cell biology-based research.

The majority of interprotein cross-links are found within the OXPHOS system. Interprotein cross-links of other mitochondrial complexes like the TOM/TIM complexes and MICOS are unfortunately rare. We also aimed to increase the yield of interprotein cross-links in such complexes by performing first protein SEC of cross-linked mitochondria under denaturing conditions followed by digestion of cross-linked protein fractions, separation of cross-linked peptides by peptide SEC and LC-MS/MS of enriched cross-links (supplemental Table S4). However, this approach did not reveal more interprotein cross-links in TOM/TIM complexes or MICOS.

We established hitherto non-described protein interactions in the IM of yeast mitochondria, which can also be used as spatial restraints for computing 3D models based on existing membrane protein complexes. Ndi1 was docked to the CIII_2_CIV_2_ supercomplex, as well as Min8 to CIV. Although all these proteins and complexes are embedded within the IM and hence are barely accessible for Lys-directed cross-linking reagents, our cross-linking data provide information about the orientation of their IMS or matrix regions, which are sufficient to propose a model for the interaction of these proteins with other membrane spanning proteins or complexes. The results obtained from the glycerol data set led us to conclude that Ndi1 - similar to CI in mammals (102, 103) - directly interacts with CIII and is part of a higher-order Ndi1_(2)_CIII_2_CIV_2_ supercomplex. Ndi1 could be identified as part of higher molecular weight respiratory supercomplexes in blue native- and clear native-PAGE experiments in combination with activity staining of NADH dehydrogenases and protein identification by MS (104–106), substantiating our model. Furthermore, CII and CV show interprotein cross-links to the CIII dimer (Fig. 4), indicating a densely packed ETC within the mitochondrial inner membrane. Bruel *et al.* (96) have already shown an interaction between CII and CIII by site-directed mutagenesis; there, mutations within the *QCR8* gene (CIII) decreased the activity of CII. Additionally, Pet9 cross-links efficiently to all complexes of the ETC, substantiating the hypothesis of an ETC-carrier supercomplex with Pet9 as a central player. In earlier studies it was demonstrated that proteins of CIII and CIV co-precipitate after affinity purification of Pet9 (91, 92). The increased number of residue pairs of Pet9 to ETC complexes in the glycerol condition are a result of glucose-repression, which reduces the copy numbers of these proteins under glucose condition (56, 57, 107).

According to the *Saccharomyces* Genome Database (16) (SGD, 04/20), 726 out of 6604 open reading frames (ORFs) are still uncharacterized. Under glucose condition we found cross-links of eleven of these proteins and under glycerol condition ten proteins (supplemental Fig. S5). Of the latter, we identified Rcf3 and Min8 cross-linked to the same amino acid of Cox12 in CIV. The localization and orientation of Min8 within the inner membrane was described recently by Morgenstern *et al.* (55), but it was not reported to be associated with CIV. Recently, Levchenko *et al.* (108) and Strecker *et al.* (109) independently identified a new supercomplex-associated protein, Cox26, which was then found to be part of the yeast CIII_2_CIV_2_ supercomplex structure by cryo-EM analyses (71). We found Cox26 cross-linked to Cox6 (Fig. 4). Interestingly, Cox26 and our modeled Min8 structure are similar with respect to protein size and helical structure. Therefore, Min8 might - similar to Cox26 be another subunit of CIV that helps to stabilize the complex. Compared with the Rcf1–3 proteins, which contain 120–224 amino acids and at least two transmembrane helices, Min8 is smaller (72 amino acids) and has only one transmembrane helix. No cross-links have been identified between Rcf2, Rcf3, and Min8 suggesting an independent function in CIV biogenesis or function. Our functional analyses on Min8 revealed that it is not essential for CIV biogenesis. However, when Min8 is lacking, the assembly of newly imported Cox12 is delayed in agreement with the structural proximity between these proteins.

An “enigma” in terms of its spatial orientation and localization represents the highly abundant but surprisingly poorly functional characterized protein Om45. Om45 has originally been described as an outer membrane protein, facing into the cytosol (87, 88), although IMS localization has also been reported (89). We found that Om45 extensively cross-links to porin, which is in accordance with a previous biochemical study (89, 110). We also show that Om45 extensively cross-links to Nde1 suggesting that it forms a complex. Because Nde1 also cross-links to Pet9 of the IM our data confirm that large parts of Om45 are localized in the IMS. Furthermore, cross-links of Om45 to the IM protein Phb1 further supports the localization of Om45 in the IMS (111, 112).

The quantitative XL-MS data reveal differences in the protein-protein network of mitochondria under glycerol *versus* glucose condition but also implies current limitations in the application of quantitative cross-linking in cellular or compartmental systems. (Quantitative) changes of cross-links among proteins in two different states are mainly because of the changes of the expression pattern. This implies that changes in the protein interactions undergone by those proteins whose expression pattern does not change under different conditions are barely detectable. Furthermore, the vast majority of intraprotein cross-links hampers detection of significant changes in interprotein cross-links. A similar observation was made in multidrug-resistant human carcinoma cells (113). More specifically for our study: the change of protein abundances caused by glucose-repression (56, 57, 107) was the main driving force of the identified differences. Accordingly, proteins that belong to the OXPHOS system or the TCA cycle have a higher copy number in mitochondria from yeast grown under glycerol than under glucose condition and hence show more intraprotein cross-links. Quantitative changes of intraprotein cross-links of proteins that are expressed equally strong under both conditions are observed for *e.g.* Pim1, Aim45, Aim46, Alo1, Arg5,6, Dld2 and Ssc1. Such cross-links might indicate conformational changes within the respective proteins depending on the growth condition. Changes of interprotein cross-links within equally expressed proteins are observed in the PDH, which might suggest a different assembly state under glucose and glycerol conditions. Such information might prove useful for structural analysis of yeast PDH.

Despite the improvement of cross-linking reagents *e.g.* MS-cleavable reagents (42, 44, 45, 114) and computational search algorithms for XL-MS (43, 46, 115–117) the analysis depth of XL-MS studies remains a challenge (118). For instance, XL-MS of murine mitochondria using a MS-cleavable cross-linker and application of improved software algorithms also identified 90% of all cross-links in the 400 most abundant proteins (48). The reasons for this are clear: the cross-linking reaction has only a certain yield on the respective amino acid residues and also bares a stochastic nature. Consequently, in less abundant proteins, in which only a low number of linear peptides are identified even in classical LC-MS/MS, cross-linked peptides are even much less abundant and hence escape analysis or might be detected only as “one-hit-wonders.” Accordingly, transient protein interactions in low abundant protein complexes are currently barely detectable by XL-MS. This issue can be solved by drastically increasing the amount of cross-linked peptides (64) to be analyzed by LC-MS/MS together with an improvement of ion filtering possibilities (*e.g.* ion mobility), increased sequencing speed and overall sensitivity of mass spectrometers in conjunction with improved computational algorithms.

## DATA AVAILABILITY

The mass spectrometry .raw data, identification lists, protein lists for fasta files and annotated spectra of cross-linked peptides have been deposited to the ProteomeXchange Consortium (www.proteomexchange.org) via the PRIDE (119) partner repository with the data set identifier PXD017620.

## Supplementary Material

Supplemental Data

Supplemental Tables
