# Supplementary material for: A Cross-linking Mass Spectrometry Approach Defines Protein Interactions in Yeast Mitochondria
Source: Mol Cell Proteomics. 2020 Apr 24;19(7):1161–78. doi: 10.1074/mcp.RA120.002028 (PMC7338081; doi:10.1074/mcp.RA120.002028)
Supplement: Supplemental Data [file 159397_1_supp_517772_q9kg1k.pdf]

Supplemental Data

A Cross-linking Mass Spectrometry Approach Defines Protein Interactions in Yeast Mitochondria

Andreas Linden[1,2], Markus Deckers[3], Iwan Parfentev[1], Ralf Pflanz[1], Bettina Homberg[3], Piotr Neumann[5], Ralf Ficner[4,5], Peter Rehling[3,4,6,*], and Henning Urlaub[1,2,*]

[1]Bioanalytical Mass Spectrometry Group, Max Planck Institute for Biophysical Chemistry, Göttingen, Germany

[2]Institute of Clinical Chemistry, University Medical Center Göttingen, Göttingen, Germany

[3]Department of Cellular Biochemistry, University Medical Center Göttingen, Göttingen, Germany

[4]Cluster of Excellence “Multiscale Bioimaging: from Molecular Machines to Networks of Excitable Cells” (MBExC), University of Göttingen, Göttingen, Germany

[5]Department of Molecular Structural Biology, Institute for Microbiology and Genetics, Göttingen Center for Molecular Biosciences, Georg-August-University Göttingen, Göttingen, Germany

[6]Max Planck Institute for Biophysical Chemistry, Göttingen, Germany

*Corresponding authors. E-mails: Peter.Rehling@medizin.uni-goettingen.de; Henning.Urlaub@mpibpc.mpg.de

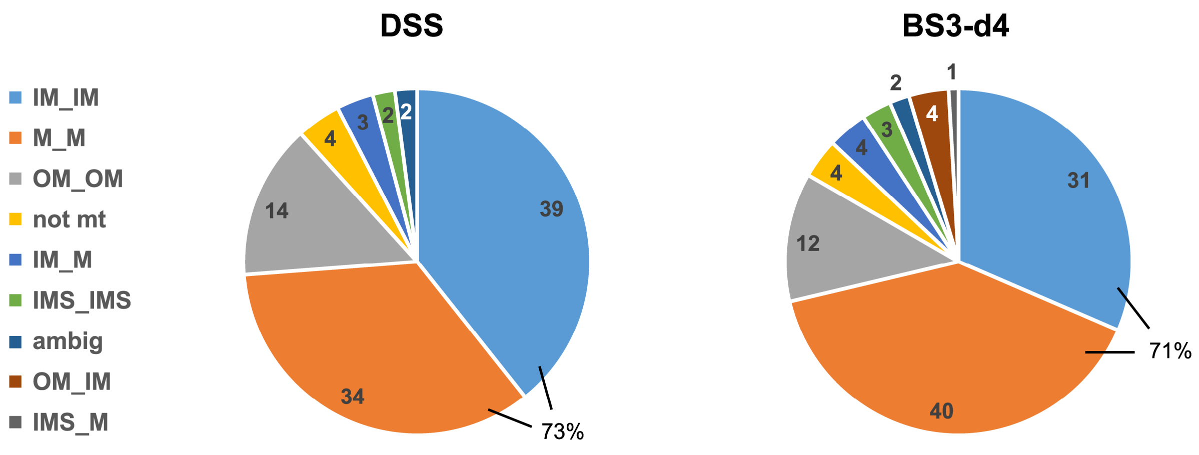

Supplemental Figure S1: Comparison of both cross-linkers DSS and BS3. To investigate if DSS and BS3 are suitable for cross-linking of mitochondria derived from yeast, DSS and isotopically labeled BS3-d4 were mixed in an equimolar ratio and added to freshly isolated crude mitochondrial extract. The pie charts illustrate the relative number of unique protein-protein interactions percent depending on the suborganellar location of the proteins after quantitative cross-linking. Quantitation was performed by XiQ (66) on identifications provided by pLink 1 (60, 61). Majority of quantified residue pairs remained unchanged. Only these protein-protein interactions with quantified residue pairs showing a fold change ≥ 2 were considered for the pie charts. OM, outer membrane; IMS, intermembrane space; IM, inner membrane; M, matrix; ambig, ambiguous; not mt, not mitochondrial.

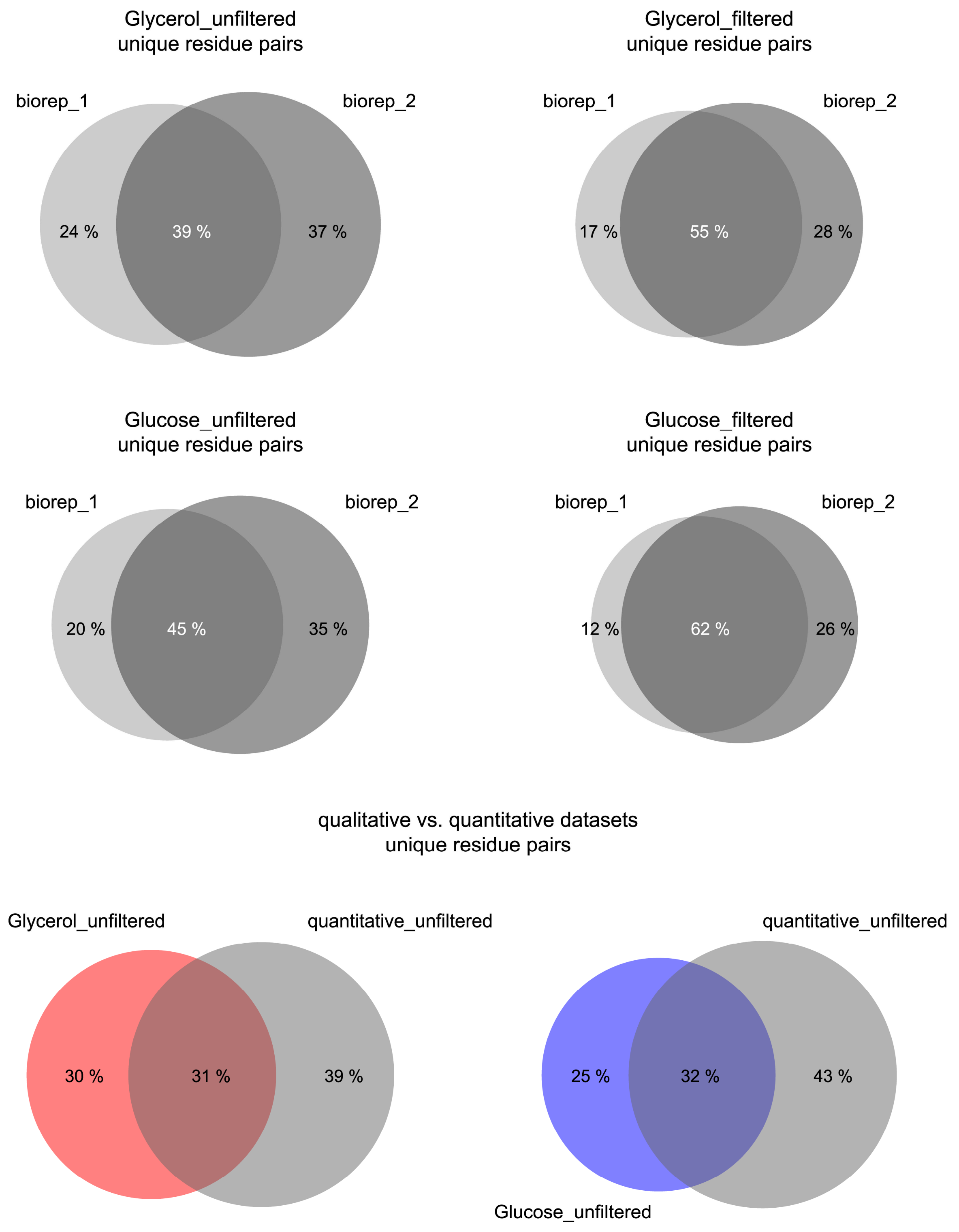

Supplemental Figure S2: Overlaps of unique residue pairs within biological replicates. Unique residue pairs from the unfiltered and filtered datasets were compared between biological replicates of the glycerol (upper panel) and glucose condition (middle panel). Unique residue pairs of all biological replicates were also compared between both the glycerol and the glucose condition and the qualitative dataset (all unfiltered).

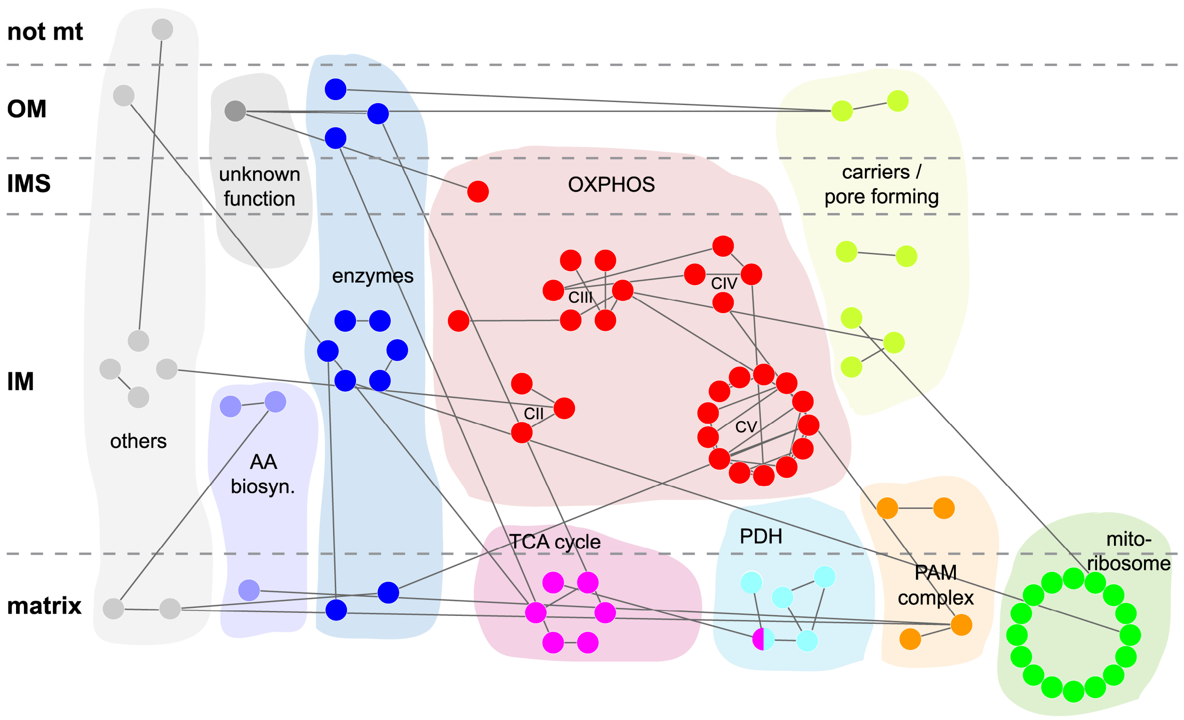

Supplemental Figure S3: Global protein-protein interaction network common under glycerol and glucose condition. Thickness of the edges is proportional to the number of unique residue pairs. OM, outer membrane; IMS, intermembrane space; IM, inner membrane; M, matrix; ambig, ambiguous; not mt, not mitochondrial; AA biosyn., amino acid biosynthesis; CoQ biosyn, Coenzyme Q biosynthesis; OXPHOS, oxidative phosphorylation system; TCA, tricarboxylic acid; PDH, pyruvate dehydrogenase complex; PAM, presequence translocase-associated motor. Networks visualized by Cytoscape (79).

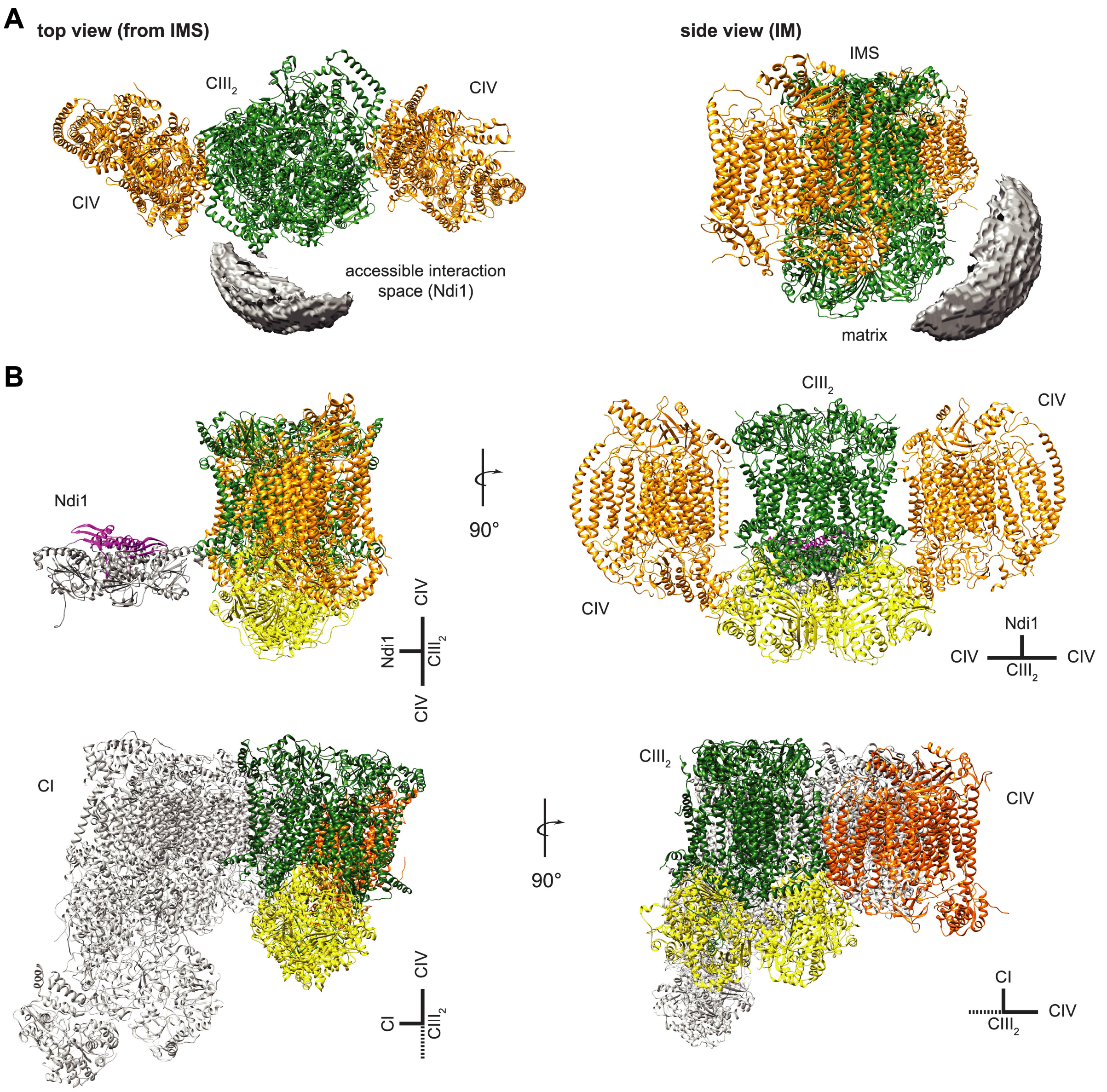

Supplemental Figure S4: Yeast and mammalian ETC supercomplex structures. *A*, Accessible interaction space of Ndi1 (gray sphere) within a Ndi1CIII$_2$CIV$_2$ supercomplex according to the identified cross-links in top view from IMS (left) and in side view within IM (right), calculated by DisVis (83). *B*, Comparison of the putative Ndi1CIII$_2$CIV$_2$ supercomplex of yeast (upper panel) with the mammalian CICIII$_2$CIV supercomplex (lower panel). Schematic T-peaces reflect the orientation of all three complexes. Green, complex III; orange, complex IV; gray, Ndi1 and complex I; light purple, membrane anchor of Ndi1; yellow, subunits Cor1 and Qcr2 of CIII; IMS, intermembrane space; IM, inner membrane. PDB CIII$_2$CIV$_2$

supercomplex: 6HU9 (71); PDB Ndi1: 4G73 (73); PDB CICIII$_2$CIV supercomplex: 5GUP (102). Structures were represented by UCSF Chimera (82).

protein	ORF	ref	cross-links to	#unique	#CSMs
**interprotein cross-links**					
Min8	YPR010C-A	*	Cox12	2	7
			Pet9	1	4
Nat2	YGR147C		Mdh1	1	2
Rcf3	YBR255C-A	**	Cox12	1	5
Ycp4	YCR004C		Hsp60	1	6
			Pst2	1	23
Aim17	YHL021C		Cpr3	1	5
Coq21	YBR230W-A	*	Coq5	1	3
Dpi8	YJL133C-A	*	Kgd1	1	34
Fmp16	YDR070C		Aco1	1	2
	YDR061W		Ilv5	2	10
**intraprotein cross-links**					
Fmp10	YER182W			1	6
Fmp41	YNL168C			1	13
Mco10	YOR020W-A			1	4
	YDR061W			1	5
	YGR266W			1	2
	YKR070W			1	2
Fmp10	YER182W			2	11
Fmp41	YNL168C			2	26
Ego4	YNR034W-A			1	7
Mco10	YOR020W-A			1	3
Mcy1	YGR012W			2	13
	YKR070W			1	24

Supplemental Figure S5: Uncharacterized proteins identified by XL-MS. List of uncharacterized proteins that showed interprotein cross-links to other proteins (top part of the table) or intraprotein cross-links (bottom part of the table). Red, identified in the glycerol dataset; blue, identified in the glucose dataset. *, described in Morgenstern *et al*. (55); **, described in Römpler *et al*. (98); #unique, number of unique cross-linked residue pairs; #CSMs, number of cross-linked peptide spectrum matches.
